# The role of TDP1 and APTX in mitochondrial DNA repair

**DOI:** 10.1016/j.biochi.2013.10.011

**Published:** 2014-05

**Authors:** Martin Meagher, Robert N. Lightowlers

**Affiliations:** The Wellcome Trust Centre for Mitochondrial Research, Institute for Cell and Molecular Biosciences, Newcastle University, The Medical School, Newcastle upon Tyne NE2 4HH, United Kingdom

**Keywords:** mtDNA, Damage, Tyrosyl-DNA-phosphodiesterase 1, Aprataxin, SSBR

## Abstract

In recent years, our knowledge surrounding mammalian mitochondrial DNA (mtDNA) damage and repair has increased significantly. Greater insights into the factors that govern mtDNA repair are being elucidated, thus contributing to an increase in our understanding year on year. In this short review two enzymes, tyrosyl-DNA-phosphodiesterase 1 (TDP1) and aprataxin (APTX), involved in mitochondrial single strand break repair (SSBR) are discussed. The background into the identification of these enzymes in mtDNA repair is communicated with further deliberation into some of the specifics relating to the import of these enzymes into the mitochondrion. With the discovery of these enzymes in mitochondria comes the probability that other mechanisms underlying mtDNA repair are yet to be fully understood, suggesting there is much left to discover when shaping our understanding of this relatively undefined subject.

## 1 Introduction

Mitochondria are organelles that are integral to viability of eukaryotes. These organelles have retained their own genome (mtDNA) in most species, which is present in multiple copies, is often maternally inherited, and in man (the focus of this review) is approximately 16.5 kb [1,2]. Mutations in mtDNA are known to accumulate during the normal ageing process, which can be accompanied by dysfunction of the machinery that couples ATP synthesis to cellular respiration (OXPHOS) [3]. For some time it was not known whether these mutations contributed to the ageing phenotype or whether these changes were just a consequence of the normal ageing process. The development of the mtDNA mutator mouse generated new and intriguing data that has since proven to be hugely influential in the understanding of the role of mtDNA mutations in ageing and disease [3,4]. While the study of mtDNA mutations is on-going and of great importance, the study of physical damage to mtDNA that may cause mutations or genome instability is a field that is still relatively undefined.

Initial investigations of mtDNA repair reported the absence of pyrimidine dimer resolution following ultra violet (UV) irradiation of mammalian cells, which may have led to the general assumption within the field that mitochondria did not possess any DNA repair capabilities [5]. This assumption remained until it was demonstrated that a variety of mechanisms exist to repair, or tolerate, mtDNA damage [6–14]. From such studies, a multitude of proteins involved in mtDNA repair have been identified [15], underlining our gross underestimation of the mtDNA repair capacity in previous years. The focus of this review will be to discuss two recently identified members of the mtDNA repair network; tyrosyl-DNA-phosphodiesterase 1 (TDP1) [12] and aprataxin (APTX) [13].

## 2 Single strand break repair in mitochondria

The process of SSBR is required in response to DNA damage caused by a multitude of sources with the most common source being reactive oxygen species (ROS) [16,17]. In such cases, bases can be damaged resulting in modification of the oxidised base leading to a single strand break (SSB), or when the oxidised based is removed through base excision repair (BER). The result is an SSB that requires ligation to restore DNA to its original form [16,17]. Mitochondria are a significant contributor to the generation of endogenous ROS. It is likely that mtDNA is susceptible to damage from endogenous ROS and that repair mechanisms are necessary as a protective measure. This has been generally accepted, as mtDNA is not covered by protective histones, but it must be mentioned that the general mtDNA binding protein TFAM is present at sufficient levels to fully coat mtDNA and promote condensation of the genome [18]. Of the repair mechanisms that have been described in mitochondria, BER is the most documented. SSBR is often considered a subpathway of BER due to end processing events to restore the 5′ and 3′ termini of the DNA to phosphate (5′-P) and hydroxyl (3′-OH), respectively, before ligation proceeds [19]. However, there are other instances whereby end processing at an SSB may occur but is not necessarily preceded by the other steps of BER i.e. removal of a damaged base and cleavage at an abasic (AP) site [16]. Such instances can include those that require the activity of TDP1 and APTX [16].

**Fig. 1:**
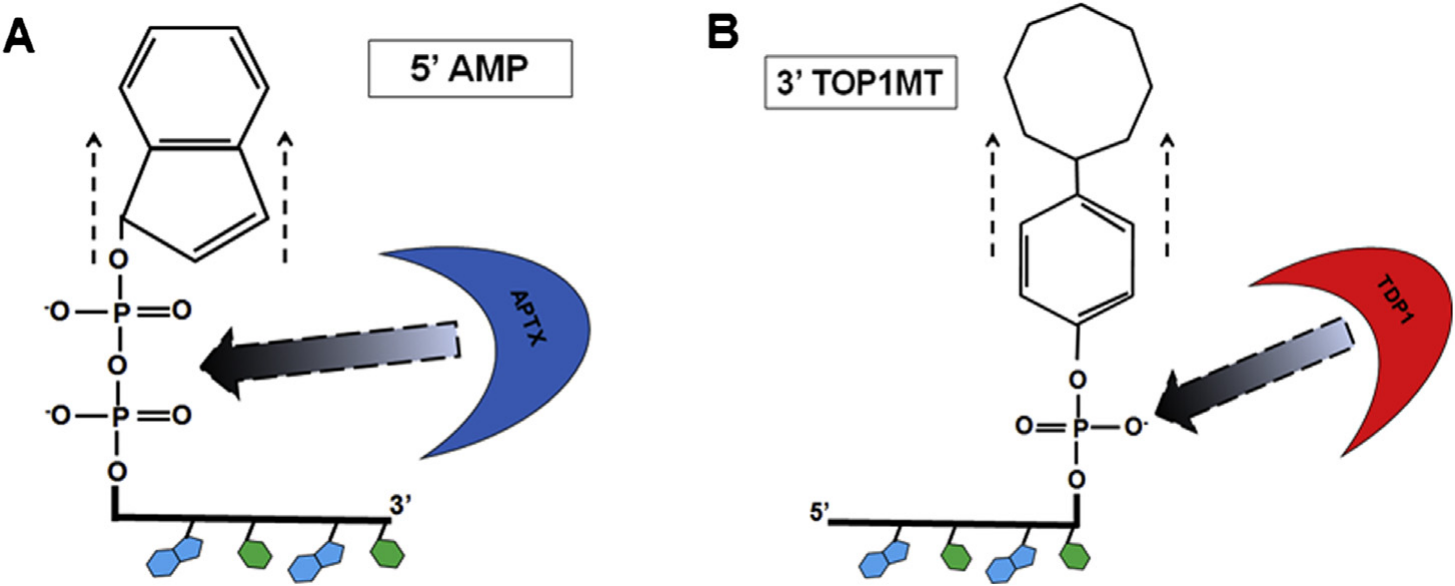
Mechanism of action of APTX and TDP1. (A) 5′-AMP lesion caused by abortive ligase activity adjacent to an existing lesion is repaired by APTX, which cleaves the pyrophosphate bond releasing AMP and leaving behind 5′-P ready for re-ligation. (B) 3′-PY generated from the collision of TOP1MT with DNA or RNA polymerases is removed by TDP1 leaving behind 3′-P that is processed further by a phosphatase restoring the mtDNA to 3′OH and primed for ligation [16].

DNA cleavage is not only initiated by ROS-induced damage. For example the mitochondrial topoisomerase (TOP1MT) ordinarily forms a transient nick in mtDNA which is thought to have a role in regulating mtDNA replication [20,21]. However, on occasion TOP1MT can collide with DNA or RNA polymerases resulting in the aberrant resolution of the DNA:TOP1MT structure, with the TOP1MT remaining covalently attached to the DNA [16,21]. Following proteolytic degradation of the attached TOP1MT, a tyrosine residue remains bound at the 3′ end of DNA via a phosphotyrosine bond (3′-PY) [16]. TDP1 is then required to resolve the 3′-PY in an end processing step that can be distinct from the other steps of BER. Recently, it has also been demonstrated that TDP1 is capable of repairing several 3′ lesions induced by chain terminating nucleoside analogues (CTNAs) [22]. Abortive ligase activity at a site adjacent to an existing lesion causes the covalent attachment of adenine monophosphate (AMP) to the 5′ end of mtDNA (5′-AMP). This unusual adduct needs to be removed by aprataxin (APTX) [16]. Fig. 1 illustrates these examples of SSBs and the mechanism of action of APTX and TDP1 to repair these lesions.

Failure to repair these lesions could have implications in genome stability as SSBs may progress to double strand breaks (DSBs), which could cause DNA instability and/or impaired replication and gene expression from the mitochondrial genome [15,16]. Mutations in *APTX* and *TDP1* have been reported to cause both ataxia with oculomotor apraxia type 1 (AOA1, for *APTX*) [23] and spinocerebellar ataxia with axonal neuropathy type 1 (SCAN1, for *TDP1*) [24] with both presenting with ataxia, a feature often, although not exclusively, associated with mitochondrial disease [25]. Due to this phenomenon, it has been suggested that both AOA1 and SCAN1 are primarily mitochondrial disorders [19,26]. However, both APTX and TDP1 activity were originally identified in the nucleus with their involvement in SSBR of nDNA being extensively studied in relation to both these diseases, and it is only recently that the presence and activity of APTX and TDP1 have been identified in the mitochondrion. This data is more consistent with both defective nuclear DNA (nDNA) and mtDNA repair contributing to the progression of each disease [26–28].

## 3 How are these enzymes imported into mitochondria?

Despite any debate over whether defective SSBR in the nucleus or mitochondrion is the primary cause of AOA1 and SCAN1, the identification of APTX and TDP1 as factors also found in mitochondria predicts novel members of the mtDNA repair network. One intriguing aspect surrounding TDP1 activity in mitochondria is how it is imported into the mitochondrion given that no N-terminal presequence is apparent, and there is little evidence to suggest a mitochondrial-specific isoform.

**Fig. 2:**
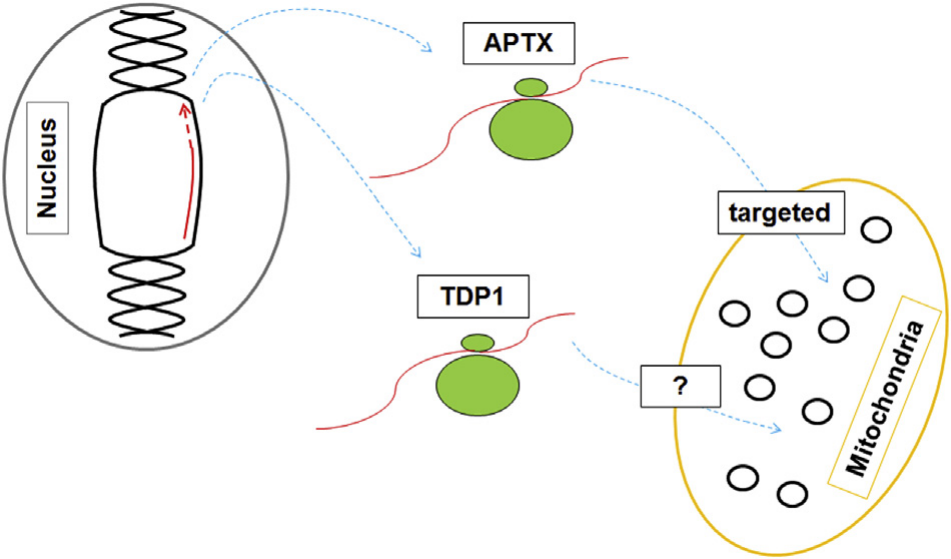
Unknown mechanism for TDP1 import into mitochondria. Illustration of mitochondrial import of APTX and TDP1 following transcription from nDNA and translation in the cytosol. APTX contains a 14-aa N-terminal MTS and therefore enters the mitochondrion by this whereas TDP1 has not been found to contain an MTS and so the mechanism by which this protein enters mitochondria has yet to be elucidated.

Unlike TDP1, an alternatively-spliced isoform of APTX was found to contain a 14 amino acid (14-aa) N-terminal sequence with a high relative potential of being a mitochondrial targeting sequence (MTS) [13], as predicted using MitoProt [29]. Similar to APTX, alternative splicing generates N-terminal targeting sequences to direct a number of other mtDNA repair enzymes to the mitochondrion, such as uracil DNA-glycosylase 1 (UNG1) and 8-oxoguanine-DNA glycosylase 1 (OGG1). This alternative splicing leads to targeting of these proteins to both nuclear and mitochondrial compartments where each isoform has a distinct molecular weight. It is often the case that in these circumstances the MTS is cleaved upon entry into the mitochondrion by a mitochondrial processing peptidase (MPP) [30]. However, with TDP1 there is no obvious N-terminal MTS despite a clear demonstration of its presence and activity in mitochondria, which suggests another import mechanism [12]. Although this is not necessarily a new conceptual problem with mitochondrial import for other proteins occurring without an N-terminal MTS it is still a relevant issue that requires addressing [31]. For the mitochondrial DNA ligase (LIG3α), import into mitochondria is achieved by providing an MTS through the use of an upstream translation initiation codon. The resultant MTS is cleaved on mitochondrial entry, such that the mature protein has the same molecular weight as in the nucleus [32]. More recently, a similar targeting scenario was discovered for the mitochondrial Flap endonuclease 1 (FEN1, or FENMIT to the authors) or the oligoribonuclease REXO2 where a ‘cryptic’ targeting signal from alternative translation initiation generates a truncated isoform of this protein that permits import [33,34]. An earlier publication from the former group based heavily on *in silico* analyses identified more than 150 putative mitochondrial proteins with similar sequences; most of which had not yet been identified in mitochondria [35]. Although quite a comprehensive list of proteins was produced from this study TDP1 was not included among them leaving the mystery surrounding the entry of this protein into mitochondria unanswered (Fig. 2).

Year on year, novel mitochondrial proteins are identified that have a variety of functions, some of which include those implicated in mtDNA repair/maintenance. The recent publications regarding the alternative translation initiation sites suggest that prediction of mitochondrial localisation for a protein maybe more complex than thought [35].

## 4 Future perspectives

When reflecting on the mechanisms of mtDNA repair; is it likely that all the major players have been identified? Almost certainly no, but to what extent does mtDNA repair, or absence of it, impact in disease and ageing? One consideration when pursuing the study of the involvement of TDP1 and APTX in mitochondria was that mutations in the genes encoding both these proteins are linked to diseases that include ataxia [16], which as stated above is a common feature of mitochondrial disease [25]. This has led to the suggestion that the mtDNA damage caused from the lack of activity of these enzymes may be the primary cause of both SCAN1 and AOA1 [26]. When considering options for investigating mtDNA repair further it may be pertinent to study enzymes that are linked to disorders with typical phenotypes of mitochondrial disease, although many mutations in genes encoding DNA repair enzymes cause a predisposition to cancer [16].

Another point for consideration with reference to mtDNA maintenance is the capability of the organelle to tolerate damage to its genome without necessarily requiring the active repair of the DNA itself. *In vitro* analyses into the ability of the mitochondrial DNA polymerase (polymerase γ) indicated that the bypass of UV-induced cyclobutane thymine dimers was possible [14]. In this study the main problem caused by these dimers was replication stalling, however, when the lesions were bypassed, misincorporation of nucleotides could be observed, suggesting a damage tolerance mechanism of repair [14]. This then suggests a mechanism of point mutation/deletion formation that is most likely preferential to replication stalling [14].

One final point with regard to the discovery of novel mtDNA repair/tolerance mechanisms in mitochondria is to echo some of the comments made by our colleagues in relation to a comparative long/short range PCR based method to measure mtDNA damage [19]. This method uses a combination of two semi quantitative PCR's and should therefore be used with caution when attempting to ascertain the exact role of an enzyme in mtDNA repair [19]. With sound analyses in future investigations into the mtDNA repair network, it may well be possible to establish the exact involvement of mtDNA damage in disease and ageing.
